# Clinical Efficacy and Safety of Pulsed Dye Laser Combined with Pingyangmycin on Hyperplastic Scar after Acne

**DOI:** 10.1155/2022/3305107

**Published:** 2022-08-28

**Authors:** Rong Guo, Wenxia Xuan, Xiao He, Kai Xu

**Affiliations:** Dermatology, Shanxi Bethune Hospital, Shanxi Academy of Medical Sciences, Tongji Shanxi Hospital, Third Hospital of Shanxi Medical University, Taiyuan, 030032 Shanxi, China

## Abstract

**Background:**

Acne is the most common chronic inflammatory disease of hair follicles and sebaceous glands in dermatology. Hyperplastic scar (HS), a very common sequelae of acne, is also the most common scar type in clinical practice.

**Objective:**

This research analyzed the clinical effectiveness and safety of pulsed dye laser (PDL) combined with pingyangmycin (PI) in the treatment of post-acne HS.

**Methods:**

One hundred and nine patients with post-acne HS admitted in June 2020 were selected and divided into a research group (*n* = 52) and a control group (*n* = 57) according to the difference in treatment methods. The efficacy, incidence of adverse reactions, skin repair, treatment comfort, and satisfaction were compared between groups.

**Results:**

The total effective rate was higher in the research group compared with the control group. No statistical difference was observed between groups in the incidence of adverse reactions. The research group showed better scar repair, skin improvement, and granulation tissue maturity than the control group. And compared with the control group, the growth factor of the research group was lower, while the treatment comfort and satisfaction, psychological state, and prognosis quality of life were higher. The two groups showed no notable difference in the recurrence rate.

**Conclusions:**

PDL combined with PI can effectively improve the clinical efficacy, scar repair effect, overall skin status, and treatment experience of patients and boost the psychological state and prognostic quality of life of patients, which has great clinical application prospect for the treatment of HS.

## 1. Introduction

Acne, mainly manifested as acne, pustules, nodules, and seborrhea, is the most common systemic chronic inflammatory disease of hair follicles and sebaceous glands in dermatology [1]. Adolescents are a high-risk group for acne, with studies indicating that more than 54% of college students have acne of varying degrees [2, 3]. Although effective results have been achieved in the treatment of acne in clinical practice, the sequelae have always been a major concern for both clinics and patients [4]. Among them, hyperplastic scar (HS), which is formed by excessive proliferation and repair of new connective tissue after tissue damage, is not only an extremely common sequelae of acne but also the most common type of scar in clinical practice [5, 6]. The survey showed that the incidence of HS in acne patients reached about 50%–55% [7, 8]. As HS cannot repair itself, once it occurs, it will accompany patients for life. Moreover, due to the existence of HS, patients generally have varying degrees of inferiority and autism, resistance and fear to communicate with others, and significantly increased risk of developing depression, autism, and other mental diseases [9, 10]. Therefore, an effective and stable HS repair program is not only a necessary means to improve the social life quality of patients but also one of the long-term hotspots of clinical skin repair.

Although there are many clinical treatments for HS at present, there is no recognized optimal therapy due to great individual differences [11]. Pulsed dye laser (PDL) therapy is a novel technology for skin diseases in recent years. According to the theory of selective photothermolysis, it penetrates the epidermis and dermis and directly into the lesion area and then selectively destroys the dilated vascular tissue, thus achieving the purpose of sealing [12]. It can also cut off the nutrient supply of blood vessels to diseased tissues and further prevent blood vessel recanalization [13]. PDL has longer wavelengths and stronger penetration ability than traditional laser and is highly safe as its specific wavelength is absorbed by only hemoglobin and blood vessels, with almost no adverse effect on the surrounding normal tissues [14]. For skin diseases such as keratosis and acne, PDL has been verified to contribute to remarkable therapeutic effects and its application in HS has been unanimously recognized [15, 16]. Pingyangmycin (PI) is a classic therapeutic drug for HS, with the primary effects of inhibiting the proliferation of fibroblasts and accelerating the atrophy and apoptosis of vascular endothelial cells [17]. Previous studies have confirmed the effectiveness of PI combined with PDL in improving the repair effect of patients with hemangioma [18], but it remains unclear how effective the combination of the two is in the treatment of HS.

Thanks to the gradual improvement of PDL, our hospital has gradually used it as the preferred treatment for HS this year, with relatively stable and remarkable results achieved by adopting it with PI together. The experimental results are reported as follows to provide more choices for future clinical therapy of HS and lay a foundation for the application of the combination of the two.

## 2. Materials and Methods

### 2.1. Data about Patients

A total of 109 patients with post-acne HS admitted to our hospital in June 2020 were enrolled and assigned to a research group (*n* = 52) and a control group (*n* = 57) according to the difference in treatment methods.

### 2.2. Inclusion and Exclusion Criteria

The inclusion criteria were as follows: patients ≥ 18 years old, patients meeting the diagnostic criteria of HS after acne, patients with skin problems defined as III–IV type according to the Fitzpatrick skin type classification [19], and those without a history of major diseases. The exclusion criteria were as follows: patients with scar constitution; photosensitive patients; pregnant or lactating patients; patients who had received surgery, hormone drugs, or laser treatment within 3 months before treatment; patients with abnormal coagulation, immunity, or organ function; and those with chronic cardiocerebrovascular diseases.

### 2.3. Methods

#### 2.3.1. Data about Instruments

A 2940 nm erbium fractional laser (MCL30, Asclepion, Germany) was used, and its treatment parameters were set as follows: laser beam: 120 *μ*m; frequency: 20 Hz; energy: 20–40 J/cm^2^; width: 100 *μ*s; and spot size: 2 mm. And the parameters of the 595 nm PDL (CADE-LA, Candela Vbeam, USA) used were as follows: frequency: 1.5 Hz; energy: 10–15 J/cm^2^; width: 1.5–2.0 ms; and spot size: 7 mm.

#### 2.3.2. Treatment Regimen

For the research group, 1 mg/mL solution, prepared by 8 mg PI (Beijing Pufei Biotechnology Co. Ltd., PC0373), 2% lidocaine injection (Chongqing Kangzhou Zhitong Pharmaceutical Technology Co. Ltd., 12MHB18), and 0.9% sodium chloride, was injected radially to the middle of the scar along its edge at 2 mL PI solution/1 cm^2^ scar. The scar turned pale after the injection. Injections, one each, were given 1 h before each PDL treatment. Patients received PDL (once a month) 1 hour after injection and erbium fractional laser treatment (once a month) one week later. The control group was treated with erbium fractional laser only once a month. Both groups were treated continuously for 4 months.

#### 2.3.3. Blood Sample Collection

The next day after each month's treatment, 5 mL fasting venous blood was sampled from each patient into coagulation-promoting tubes and centrifuged to collect serum, to quantify its transforming growth factor-beta 1 (TGF-*β*1) and bone morphogenetic protein-7 (BMP7) by ELISA [20]. The kits were all ordered from Jiangxi Aiboyin Biotechnology, and all operations were carried out under the instructions.

#### 2.3.4. Follow-Up for Prognosis

After the whole treatment, each patient was followed up once every three months for one year for prognosis judgment. The follow-up was conducted in the form of telephone notification for hospital review.

### 2.4. Efficacy Evaluation

Markedly effective is scar repair area > 75%; effective is scar repair area between 50% and 75%; moderate is scar repair area between 25% and 50%; ineffective is scar repair area < 25%. Total effective rate = (the number of patients with markedly effective treatment + the number of patients with effective treatment)/the total number of patients × 100%.

### 2.5. Outcome Measures

1. Clinical efficacy
2. Safety: the incidence of adverse reactions during treatment was recorded
3. Scar repair: indicators including the scar area and thickness before and after treatment, Vancouver scar scale (VSS) score [21], and visual score of scar (score range: 0–6 points; the scar was scored according to its visibility and visible distance, with higher scores indicating more obvious scar) were assessed
4. Skin improvement: indicators including VISIA scores of skin texture and pore before and after treatment were recorded. The VISIA skin analysis system was used, and the test results were presented as percentages, with higher percentages indicating better skin condition
5. Maturation ability of granulation tissue: after the first (T1), second (T2), third (T3), and fourth months (T4) of treatment, the grade of granulation tissue maturity in scar was evaluated. In grade IV, the wound was completely covered by granulation tissue; in grade III, the wound was not completely covered by granulation tissue, but the covered area exceeded one half of the total area; in grade II, the wound was covered by granulation tissue by no more than a half; in grade I, the wound surface showed new granulation tissues; in grade 0, there was no new granulation tissue in the wound
6. TGF-*β*1 and BMP-7 levels were detected by immunohistochemistry (SP method) from T1 to T4
7. Comfort: the visual analog scale (VAS), numerical rating scale (NRS) for itching, and general comfort questionnaire (GCQ) scores were used for comfort assessment of patients. Among them, the VAS (score range: 0–10 points, with 0, 1–3, 4–6, and 7–10 points indicating no, mild, moderate, and severe pain, respectively) and NRS (score range: 0–10 points, with 0 indicating no itching and 10 indicating worst itching) scores were positively correlated with pain and itching, respectively, while the GCQ was scored on a 4-point Likert scale with a total score of 28–112 points and higher scores were associated with more obvious comfort
8. Psychological state: we have the self-rating anxiety scale (SAS) (50–60, 61–70, and 70 points above indicated mild, moderate, and severe anxiety, respectively) and self-rating depression scale (SDS) (53–62, 63–72, and 73 points above indicated mild, moderate, and severe depression, respectively) scores before and after treatment
9. Treatment satisfaction: after treatment, the treatment satisfaction was surveyed with a total score of 100 points. A score lower than 60 points indicated dissatisfaction, a score between 60 and 85 points indicated satisfaction, and a score above 85 points indicated high satisfaction. Overall satisfaction = (the number of patients with high satisfaction + the number of those with satisfaction)/the total number of patients × 100%
10. Prognostic life quality: an acne-specific scale was adopted to evaluate patients' life quality from the domains of social function, acne symptoms, emotional function, and self-cognition. The scores of the four subscales were presented independently, with a minimum clinical difference of 2 points and a range of 0–30 points. A higher score indicated higher life quality
11. Recurrence: recurrence rates of acne and HS after treatment were calculated

### 2.6. Statistical Analyses

This study adopted SPSS23.0 for analyses of all data. Intergroup comparisons of enumeration data presented by (*n*[%]) were conducted via the chi-square test, while intergroup comparisons of measurement data, presented by the ($\bar{\chi }\pm s$), were performed by the independent samples *t*-test, one-way ANOVA, and LSD post-hoc test. *P* < 0.05 denotes a significant difference.

## 3. Results and Discussion

### 3.1. The Two Groups Are Not Statistically Different in Clinical Baseline Data

As shown in Table 1, the two groups were not statistically different in clinical baseline data (all *P* > 0.05).

### 3.2. The Research Group Has Obtained a Higher Clinical Efficacy than the Control Group

As shown in Table 2, the total effective rate in the research group was 90.38%, higher than that in the control group (75.44%) (*P* < 0.05).

### 3.3. The Two Groups Are Not Different in the Incidence of Adverse Reactions

As shown in Table 3, the incidence of adverse reactions in the research group was not different from that in the control group (17.31% vs. 14.04%, *P* > 0.05).

### 3.4. The Scar Repair in the Research Group Is Better than That in the Control Group

**Figure 1:**
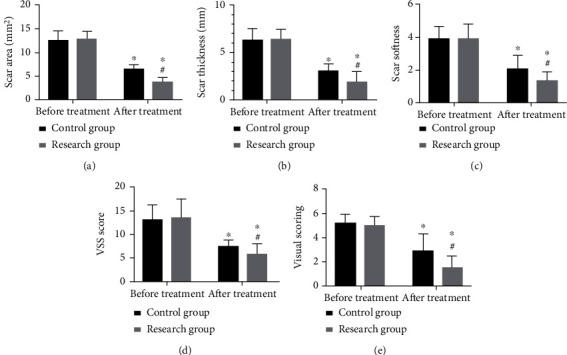
Comparison of scar repair. (a) Scar area before and after treatment. (b) Scar thickness before and after treatment. (c) Scar softness before and after treatment. (d) VSS score before and after treatment. (e) Visual score before and after treatment. ^∗^Difference vs. the situation before treatment. ^#^Difference vs. the control group. VSS: Vancouver scar scale.

Before treatment, no statistical difference was found between the two groups in the scar area and thickness (both *P* > 0.05), while after it, scar thickness, area, softness, VSS score, and visual score in the research group were all lower than those in the control group (all *P* < 0.05) (Figure 1).

### 3.5. The Overall Skin Improvement in the Research Group Is Better than That in the Control Group

**Figure 2:**
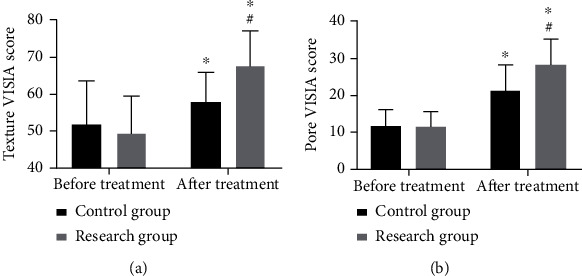
Comparison of skin improvement. (a) VISIA score of skin texture before and after treatment. (b) VISIA score of skin pores before and after treatment. ^∗^Difference vs. the situation before treatment. ^#^Difference vs. the control group.

Before treatment, the two groups were not statistically different in VISIA scores of texture and pores (both *P* > 0.05), while after it, the scores in the research group were significantly higher (both *P* < 0.05) (Figure 2).

### 3.6. The Granulation Tissue Maturation in the Research Group Is Higher than That in the Control Group

At T1 and T4, the two groups were not statistically different in the maturation ability of granulation tissue (both *P* > 0.05), but at T2, the granulation tissue maturation was higher in the research group (*P* < 0.05). In addition, at T3, no difference was found in the overall granulation tissue maturation between the two groups (grade I + grade II + grade III + grade IV) (*P* > 0.05) but higher grade IV maturation was determined in the research group compared with the control group (*P* < 0.05) (Table 4).

### 3.7. The Research Group Presented Lower Growth Factor Levels than the Control Group

**Figure 3:**
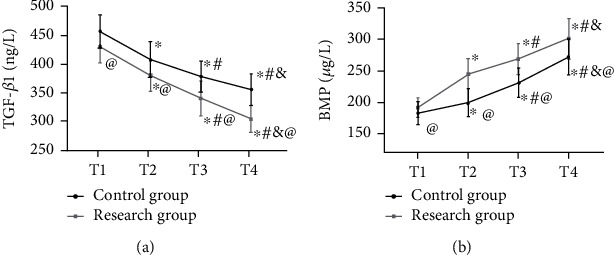
Comparison of growth factors. (a) TGF-*β*1 during treatment. (b) BMP-7 during treatment. ^∗^Difference vs. the T1. ^#^Difference vs. the T2; ^&^difference vs. the T3; ^@^difference vs. the control group. TGF-*β*1: transforming growth factor-beta 1; BMP-7: bone morphogenetic protein-7.

The research group showed lower TGF-*β*1 and higher BMP-7 than the control group from T1 to T4 (*P* < 0.05). In both groups, TGF-*β*1 reached the highest at T1, decreased at T2, and reached lowest at T4, while BMP-7 showed the opposite trend (all *P* < 0.05) (Figure 3).

### 3.8. The Research Group Experienced Higher Treatment Comfort than the Control Group

**Figure 4:**
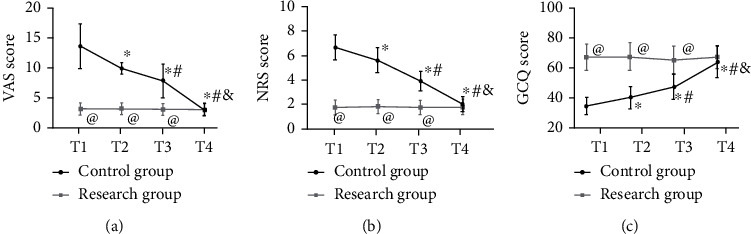
Comparison of comfort. (a) VAS score during treatment. (b) NRS score during treatment. (c) GCQ score during treatment. ^∗^Difference vs. the T1. ^#^Difference vs. the T2; ^&^difference vs. the T3; ^@^difference vs. the control group. VAS: visual analogue scale; NRS: numerical rating scale; GCQ: general comfort questionnaire.

At T4, VAS, NRS, and GCQ scores of the two groups were not statistically different (all *P* > 0.05), while at T1, T2, and T3, the research group presented lower VAS and NRS scores and higher GCQ scores than the control group (all *P* < 0.05). In addition, from T1 to T4, there was no notable change in the three scores in the research group (*P* > 0.05), while the VAS and NRS scores of the control group reached the highest at T1, began to decrease at T2, and reached the lowest at T4 and the situation of GCQ score was opposite (all *P* < 0.05) (Figure 4).

### 3.9. The Research Group Had Better Psychological State than the Control Group

**Figure 5:**
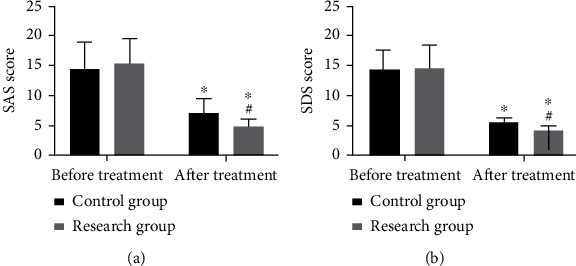
Comparison of psychological state. (a) SAS score before and after treatment. (b) SDS score before and after treatment. ^∗^Difference vs. the situation before treatment. ^#^Difference vs. the control group. SAS: self-rating anxiety scale; SDS: self-rating depression scale.

Before treatment, no difference was found in psychological state-associated scores (all *P* > 0.05), while after it, the research group got lower SAS and SDS scores than the control group (both *P* < 0.05) (Figure 5).

### 3.10. The Research Group Expressed Higher Treatment Satisfaction than the Control Group

The treatment satisfaction of the research group was higher than that of the control group (92.31% vs. 78.95%, *P* < 0.05, Table 5).

### 3.11. The Research Group Had Higher Prognostic Life Quality than the Control Group

**Figure 6:**
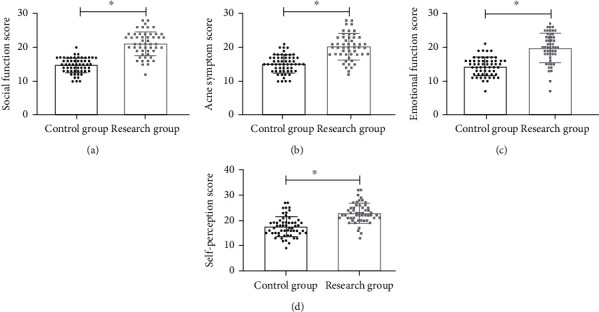
Comparison of prognostic life quality. (a) Social function score. (b) Acne symptom score. (c) Emotional function score. (d) Self-perception score. ^∗^There is a difference between the two groups.

We successfully followed up 50 patients in the research group and 56 patients in the control group for one year. The scores of social function, acne symptoms, emotional function, and self-perception of the research group were found to be all higher than those of the control group (all *P* < 0.05, Figure 6).

### 3.12. The Two Groups Were Not Greatly Different in Recurrence Rates

**Figure 7:**
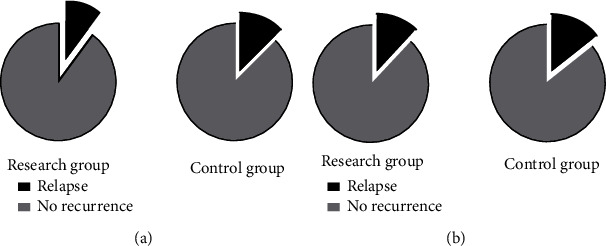
Comparison of recurrence rates.

The recurrence rates of acne and HS in the research group were 10.00% and 12.00%, respectively, while those in the control group were 12.5% and 14.29%, respectively, showing no statistical difference between the two groups (both *P* > 0.05, Figure 7).

### 3.13. Selection of the Optimal Treatment Time for HS

According to the median course of disease in the research group (12.13 months), the patients were assigned to either the short-course (<12.13 months) group or the long-course (≥12.13 months) group. The total effective rate of the short-course group was higher than that of the long-course group (*P* < 0.05, Table 6).

### 3.14. Discussion

Acne, a skin condition with a high incidence among young and middle-aged people, poses a huge negative impact on the normal life of patients [22]. As a high-incidence sequela after therapy of acne, HS has also captured close clinical attention [23]. How to improve the repair of HS is the focus and difficulty of clinical research over the past few years [24]. The advancement of PDL technology in recent years has laid a foundation for breakthroughs in HS treatment [25]. However, there are few research data on PDL combined with PI in the treatment of HS, so this study is aimed at providing a reference for this combined treatment plan and at providing new ideas for the treatment of HS in the future.

First of all, we compared the clinical efficacy between the two groups. According to the results, the research group obtained a higher total efficacy than the control group, suggesting the remarkable effect of PDL combined with PI on HS. Erbium fractional laser is a nonstripping fractional laser based on the principle of fractional photothermolysis. A columnar microtreatment area can be formed in the dermal layer by dividing the laser into several discontinuous microspots and allowing them to penetrate the skin surface [26]. In the treatment area, water molecules absorb the laser light and produce a thermal energy response that spreads further in the cortex, activating keratinocytes to repair damaged epidermis [27]. Moreover, the thermal effect can promote the formation of neocollagen in the dermis and further promote the repair of scars [28]. In the erbium fractional laser therapy, water is utilized to treat the target tissue. Studies have confirmed that water molecules cannot absorb erbium laser wavelength and the stratum corneum contains very little water, so erbium fractional laser will not destroy the normal tissue of the dermis [29, 30]. Coagulation necrosis occurs only in the treatment area during the application process, so the erbium fractional laser therapy will not cause exfoliative stomata that is found in traditional laser treatment such as CO_2_ fractional laser. Moreover, it protects the complete structure and functionality of stratum corneum, accelerates injury repair, and improves the treatment experience of patients [31, 32]. Because of its stable efficacy and safety, erbium fractional laser is increasingly applied in the treatment of HS and is also the first choice for HS therapy in most cases [33, 34]. However, during its increasing widespread application, its limitations have also been exposed. For example, Hui et al. [35] proposed that erbium fractional laser should be combined with autologous platelet-rich plasma and anemic platelet plasma to alleviate skin aging.

El-Taieb et al. [36] have pointed out that erbium fractional laser is not ideal for atrophic scar as a single treatment scheme. Thanks to the improvement of PDL technology in recent years, a new direction has emerged in the therapy of HS. Like erbium fractional laser, PDL also contributes to HS treatment through pore expansion based on selective photothermolysis [37]. However, PDL inhibits the secretion of sebaceous glands in the process of promoting the expulsion of keratinized epithelium and reducing inflammatory substances in hair follicles, which can not only alleviate the inflammatory reaction of skin tissue but also regulate the arrangement of dermal fibroblasts and promote the repair and regeneration of scar tissue [38]. Therefore, in addition to scar itself, PDL can also improve the skin tissue around the scar. PI, as a bleomycin antitumor antibiotic produced by pingyang streptomycin, can suppress DNA synthesis and cut off the DNA chain in abnormally exuberant proliferating cells [39]. In addition, as a cell cycle nonspecific drug, PI has no negative impact on the immune function and hematopoietic function of the body, with well-documented drug safety [40]. As we all know, HS is attributed to the formation of new skin tissues due to abnormal proliferation of endothelial cells after skin tissue injury and destruction [41]. PI can destroy collagen cells and promote collagen dissolution to inhibit the proliferation of vascular endothelial cells and reduce the possibility of new scar formation as well as the blood supply to scar tissue, thus facilitating the transformation and repair of scar [42]. During its application in combination with PDL, PI can slow down the abnormal proliferation of vascular endothelial cells caused by cell metabolism after laser treatment and pulsed dye can be directly transmitted to the dermal tissue through thermal effect and dermal micropore-enhanced PI effect to enhance the efficacy of PI. The synergy of the two not only reduces the risk of treatment but also enhances the repair effect. We inferred that the synergistic action was also the leading reason for the higher clinical efficacy in the research group compared with the control group. The study by Zhao et al. [43] has also revealed the more remarkable effect of PI combined with sodium houttuyfonate on treating facial venous malformation, which could also preliminarily support our experimental results. Second, the insignificant difference between the two groups in the incidence of adverse reactions also fully demonstrated the relatively high safety of PDL combined with PI and its application value in clinical practice. Then, we compared the scar and skin repair between the two groups. The results also revealed better repair effect on the research group compared with the control group, which further verified our above inference. The observation of granulation tissue maturation during treatment revealed that the new granulation tissue in the research group basically matured after 3 months of treatment, which suggested that PDL combined with PI could effectively shorten the treatment cycle of patients. In terms of the reason, we speculated that PDL combined with PI promoted the secretion of low molecular peptides on the skin surface and accelerated the proliferation and division of cells, thus promoting the regeneration of epithelial and mucosal tissues and finally creating a favorable environment for scar repair [44]. This can be verified by our detection results of TGF-*β* and BMP-7 in the two groups. Reportedly, TGF-*β*1, as a representative substance of fibrosis-promoting factors, elevates abnormally in the process of scar formation [45]. While BMP-7 is a crucial member of the TGF-*β* superfamily that can participate in collective metabolism by binding with receptors, the deletion of BMP-7 receptors in cell membrane will lower the inhibitory effect of TGF-*β*1 on fibrosis by negatively regulating Smad protein [46]. The decrease in TGF-*β*1 and increase in BMP-7 in the research group fully demonstrated that the ability of skin tissue fibrosis was greatly reduced in the group. We also compared patient comfort during treatment between the two groups and found that the research group experienced milder pain and itching and stronger comfort than the control group. Patients will experience varying degrees of discomfort due to photothermal effect in the initial stage of laser therapy, and the milder skin burning pain caused by photothermal effect in the research group may be explained by the preuse of PI and pulse dye. As mentioned above, HS has the most severe negative effect on the psychological state of patients. Thus, in HS treatment, efforts should be made to repair the scar while improving the psychological state of patients. In our study, the research group had statistically lower SAS and SDS scores after treatment, suggesting that PDL combined with PI could also effectively improve patients' psychological state and enhance their prognosis and self-confidence in communicating with others. The results were also reflected in our follow-up survey on patients' satisfaction and prognostic life quality, which fully demonstrated the great application value of PDL combined with PI. Finally, by comparing the efficacy of HS patients with different courses of disease, we found that patients with a course of disease > 12 months obtained better efficacy. The reason behind it may be due to more stable and better effects of the laser treatment for immature scars, similar to the research results obtained by Kant et al. [47].

In addition to the treatment of post-acne HS, PDL combined with PI is also highly effective for scars caused by burns and surgery [18], which is one of our follow-up research directions. In addition, in this study, only erbium fractional laser has been used as a control and the effect of PDL combined with PI may not be as significant as expected when compared with other schemes. Moreover, we need to carry out *in vitro* experiments as soon as possible to confirm the therapeutic mechanism of PDL combined with PI on HS.

## 4. Conclusion

PDL combined with PI is effective and safe in the treatment of HS and can effectively improve the scar repair and treatment experience of patients, which is worth popularizing in clinical practice.

## Figures and Tables

**Table 1:** Comparison of clinical baseline data.

	Research group	Control group	*t* or *χ*^2^	*P*
Age	23.46 ± 3.01	23.09 ± 3.06	0.636	0.527
Course of disease (months)	12.13 ± 4.35	11.71 ± 4.14	0.516	0.607
Gender			0.243	0.622
Male/female	28/24	28/29		
Scar degree			0.257	0.8793
Mild/moderate/severe	12/31/9	11/35/11		
Scar site			0.517	0.772
Face/torso/limbs	34/12/6	40/10/7		
Family history of illness			0.517	0.472
Yes/no	12/40	10/47		
Smoking			0.226	0.635
Yes/no	25/27	30/27		
Place of residence			0.056	0.813
Urban/rural	42/10	45/12		
Nationality			0.445	0.505
Han/minority	50/2	56/1		

**Table 2:** Comparison of clinical efficacy [*n*(%)].

	Markedly effective	Effective	Moderate	Ineffective	Total effective rate
Research group	32 (61.54)	15 (28.85)	4 (7.69)	1 (1.92)	90.38%
Control group	23 (40.35)	20 (35.09)	8 (14.04)	6 (10.53)	75.44%
*χ* ^2^					4.220
*P*					0.040

**Table 3:** Comparison of the incidence of adverse reactions [*n*(%)].

	Burning sensation	Skin erythema	Edema	Depigmentation	Infection	The total incidence
Research group	1 (1.92)	3 (5.77)	3 (5.77)	2 (3.85)	0 (0.00)	17.31%
Control group	3 (5.26)	3 (5.26)	1 (1.75)	0 (0.00)	1 (1.75)	14.04%
*χ* ^2^						0.221
*P*						0.638

**Table 4:** Comparison of maturation ability of granulation tissue.

		0 grade	I grade	II grade	III grade	IV grade
T1	Research group	57 (100.0)	0 (0.0)	0 (0.0)	0 (0.0)	0 (0.0)
	Control group	52 (100.0)	0 (0.0)	0 (0.0)	0 (0.0)	0 (0.0)

T2	Research group	30 (52.63)	16 (28.07)	8 (14.04)	3 (5.26)	0 (0.0)
	Control group	17 (32.69)^∗^	21 (40.38)	7 (13.46)	7 (13.46)	0 (0.0)

T3	Research group	8 (14.04)	6 (10.53)	10 (17.54)	18 (31.58)	15 (26.32)
	Control group	5 (9.62)	2 (3.85)	5 (9.62)	12 (23.08)	28 (53.85)^∗^

T4	Research group	0 (0.0)	0 (0.0)	0 (0.0)	14 (24.56)	43 (75.44)
	Control group	0 (0.0)	0 (0.0)	0 (0.0)	7 (13.46)	45 (86.54)

^∗^Difference vs. the control group.

**Table 5:** Comparison of treatment satisfaction [*n*(%)].

	High satisfaction	Satisfaction	Dissatisfaction	Overall satisfaction
Research group	35 (67.31)	13 (25.00)	4 (7.69)	92.31%
Control group	22 (38.60)	23 (40.35)	12 (21.05)	78.95%
*χ* ^2^				3.876
*P*				0.049

**Table 6:** Comparison of clinical efficacy between the short-course group and the long-course group.

	Markedly effective	Effective	Moderate	Ineffective	Total effective rate
Short-course group	10 (41.67)	11 (45.83)	2 (8.33)	1 (4.17)	87.50%
Long-course group	22 (78.57)	4 (14.29)	2 (7.14)	0 (0.00)	92.86%
*χ* ^2^	17.090				0.427
*P*	<0.001				0.514

## Data Availability

The labeled dataset used to support the findings of this study is available from the corresponding author upon request.
